# Expression Status and Prognostic Significance of Gamma-Glutamyl Transpeptidase Family Genes in Hepatocellular Carcinoma

**DOI:** 10.3389/fonc.2021.731144

**Published:** 2021-08-26

**Authors:** Shan Tian, Jiao Li, Yingyun Guo, Weiguo Dong, Xin Zheng

**Affiliations:** ^1^Department of Infectious Diseases, Union Hospital, Tongji Medical College, Huazhong University of Science and Technology, Wuhan, China; ^2^Department of Gastroenterology, Renmin Hospital of Wuhan University, Wuhan University, Wuhan, China

**Keywords:** gamma-glutamyl transpeptidase, family genes, hepatocellular carcinoma, survival analysis, biomarker

## Abstract

**Purpose:**

Gamma-glutamyl transpeptidase (GGT) family genes play crucial roles in the formation and progression of several solid tumors. However, the expression patterns and the prognostic significance of GGT members in hepatocellular carcinoma (HCC) remain unknown. This study was designed to determine the expression profiles of GGT family members in HCC and validate the prognostic value of serum GGT protein in patients with HCC.

**Method:**

We comprehensively searched public resources based on the LIHC dataset to determine the expression patterns, prognostic significance, DNA methylation status, immune infiltration, and biological pathways of GGT family genes in HCC. Subsequently, we validated the prognostic value of serum GGT protein in 85 patients with early-stage HCC subjected to curative hepatectomy from the Renmin Hospital of Wuhan University.

**Results:**

Except for *GGT1*, other GGT family members (*GGT5*, *GGT6*, and *GGT7*) were found to be differentially expressed in primary HCC samples (N = 371) and normal control tissues (N = 50). Furthermore, a positive relationship was not only observed between *GGT1* and *GGT5* (Spearman coefficient: 0.24, P = 5.143 × 10^−6^) but also between *GGT5* and *GGT6* (Spearman coefficient: 0.38, P = 1.24 × 10^−13^). The expression of *GGT1*, *GGT5*, and *GGT7* was correlated with overall survival (OS), and *GGT7* was associated with disease-free survival (DFS) in patients with HCC. Negative associations between DNA methylation and expression of mRNA were observed for *GGT1* (Spearman coefficient: −0.38, P = 6.24e-14), *GGT6* (Spearman coefficient: −0.29, P = 1.23e-8), and *GGT7* (Spearman coefficient: −0.34, P=6.7e-11). GGT family genes were well correlated with the infiltration levels of immune cells in HCC, especially CD4+ T cells, macrophages, and dendritic cells. Finally, when validated with clinical data from the Renmin cohort, a high expression of serum GGT protein was identified as a strong prognostic element of unfavorable OS (HR = 3.114, P = 0.025), but not of DFS (HR = 1.198, P = 0.05) in patients with HCC subjected to curative hepatectomy.

**Conclusion:**

To our knowledge, this is the first comprehensive analysis of the expression patterns and clinical value of GGT family genes in patients with HCC. Our study laid the foundation for the clinical application of the GGT protein in the survival assessment of patients with HCC.

## Introduction

Liver cancer is the fourth leading cause of cancer-related mortalities worldwide, after lung cancer, colorectal cancer, and stomach cancer (1). Hepatocellular carcinoma (HCC) is the most common pathological type of primary liver cancer, followed by cholangiocarcinoma. The major risk factors of HCC include viral hepatitis (hepatitis B virus, hepatitis C virus), metabolic factors (non-alcoholic fatty liver disease), behavioral factors (alcoholism, frequent smoke), and aflatoxins. Although considerable achievements have been attained in the field of anti-HCC, including surgical removal, molecular targeted therapy, and chemoradiotherapy, the long-term survival outcomes of individuals with HCC have not met our expectations owing to early metastasis (2). Therefore, identifying serum biomarkers that are specific to the survival outcomes of patients with HCC is quite desirable.

α-Fetoprotein (AFP) has long been recognized as a diagnostic biomarker for HCC but was recently excluded from the surveillance criteria of several authoritative HCC management consensus (3, 4). As the overexpression of AFP is only observed in approximately 50% of patients with HCC, identifying other serum biomarkers is imperative. Gamma-glutamyl transpeptidase (GGT) is a crucial liver enzyme involved in glutathione metabolism and is responsible for the extracellular cleavage of glutathione (5). GGT is a common liver enzyme located on the surface of most cells but is the most commonly found in hepatocytes (6). GGT is routinely utilized in clinical practice to assist clinicians in identifying the presence of liver injury. However, increased serum GGT is reported to be associated with metabolic syndrome (7), chronic kidney disease (8, 9), dementia (10, 11), and even malignant tumors (12). Recently, GGT has been demonstrated as the hallmark of oxidative stress; this enzyme can induce pro-oxidant reactions, which play an essential role in tumor formation and cell proliferation (13). Although several clinical studies have revealed the association between serum GGT and overall survival (OS) in patients with HCC (14–17), the overall prognostic effect of serum GGT remains uncertain in patients with HCC. Moreover, the expression profiles of GGT family genes in liver tissues and the prognostic values of GGT family genes in patients with HCC have never been investigated. Hence, the primary goal of this study was to investigate the expression profiles, prognostic role, DNA methylation, immune infiltration, and potential biological pathways of GGT family members in HCC *via* mining data from publicly accessible datasets. The second aim was to validate the prognostic value of serum GGT protein in patients with HCC based on our cohort.

## Methods

### Clinical Data From Renmin Hospital of Wuhan University

We retrospectively gathered clinical data of patients with early-stage HCC (TNM I–II) from Renmin Hospital of Wuhan University from 2012 to 2016. The inclusion criteria were (1) patients with early-stage HCC (2), patients that underwent curative resection, and (3) patients who were tested for serum GGT protein. The exclusion criteria were (1) patients with cholangiocarcinoma (2), patients with other malignant tumors (3), patients without follow-up data, and (4) patients with advanced TNM stage HCC (TNM III–IV). The detailed clinicopathological data obtained from the Electronic Medical Record System were age at diagnosis, gender, levels of serum GGT, histological stage, tumor size, TNM stage, HBV infection, and serum AFP. All patients with HCC in our cohort were required to complete regular follow-ups after surgical resection. Patients with HCC who did not visit our outpatient department as scheduled were called for follow-ups to determine the living or recurrent status. OS and disease-free survival (DFS) were recorded during the follow-up. Based on the above criteria, 85 cases with primary HCC from the Renmin cohort were finally included in our analysis. Our study plan was strictly screened by the Ethical Committee of Renmin Hospital of Wuhan University (No. WDRY-2019-K104).

### UALCAN

UALCAN (http://ualcan.path.uab.edu) is an interactive and comprehensive web tool for analyzing cancer OMICS data (TCGA, CPTAC, and MET500) that provide users with easy access to public cancer data (18). In this study, we employed this database *via* its methylation module to gain information about the differential expression of four GGT family genes and the methylation status between liver cancerous tissues and pathological normal tissues.

### GEPIA

GEPIA (http://gepia.cancer-pku.cn) is a newly developed webpage for analyzing the RNA sequencing expression data from the GTEx and TCGA projects (19). GEPIA provides users with various functions, including differential expression analysis, survival analysis, and correlation analysis. We utilized the survival module *via* the GEPIA webpage to assess the associations between GGT family genes (*GGT1*, *GGT5*, *GGT6*, and *GGT7*) and survival outcomes (OS and DFS) of patients with HCC.

### cBioPortal

The cBioPortal for Cancer Genomics, a very powerful database (http://www.cbioportal.org/), is designed to provide consumers with visual analysis of large-scale cancer genomic datasets (20). This powerful database was employed to determine the association between two GGT family members and the correlation between DNA methylation and mRNA expression of the GGT family genes. Moreover, this genetic database was used to investigate the correlations between the expression of GGT family genes and copy number, as well as mutations.

### UCSC Xena

UCSC Xena (http://xena.ucsc.edu), designed by the University of California–Santa Cruz, is an online exploration webpage for visualizing tumor data (21). We used this database to retrieve the clinical information and RNA-sequencing data of GGT family genes in TCGA-LIHC dataset. This powerful database was also employed, *via* its visualization module, to determine the distribution of the CpG sites of four GGT family genes in HCC.

### TIMER Database

TIMER database (https://cistrome.shinyapps.io/timer/) is a comprehensive resource for systemic analysis of six immune infiltrates (CD4+ T cells, B cells, CD8+ T cells, macrophages, neutrophils, and dendritic cells) across diverse cancer types (22). TIMER database allows users to freely input specific parameters, resulting in a picture display to conveniently assess tumor immunological, clinical, and genomic features. We utilized the gene module to determine the correlations between four GGT family genes and immune infiltrates in HCC.

### Gene Set Enrichment Analysis

The gene expression values of the LIHC dataset were downloaded from TCGA database (https://www.cancer.gov/). All individuals with primary HCC were divided into high- or low-expression group based on the median value of *GGT1*, *GGT5*, *GGT6*, and *GGT7*. Thereafter, the cluster profiler R package (https://www.r-project.org) in R software (version 3.0) was employed to identify the most likely biological pathways in HCC.

### Statistical Analysis

Whole data analysis was implemented with SPSS software (version 21.0) and GraphPad Prism (version 7.0). *GGT1*, *GGT5*, *GGT6*, and *GGT7* were transformed into categorical variables (low expression or high expression) according to the median value. Serum GGT protein and serum AFP were also transformed into categorical variables (normal concentration or high concentration) based on the reference value of Renmin Hospital of Wuhan University. The difference in serum GGT levels between two groups was determined by the Student’s t test. For analysis of survival, the log-rank test was used to compare the median survival time between two groups. Multivariate analysis combined with univariate surviving analysis was conducted to identify the independent prognostic factor in patients with HCC. A two-tailed P value less than 0.05 was regarded as statistically significant.

## Results

### Expression Status of GGT Family Genes in HCC

**Figure 1:**
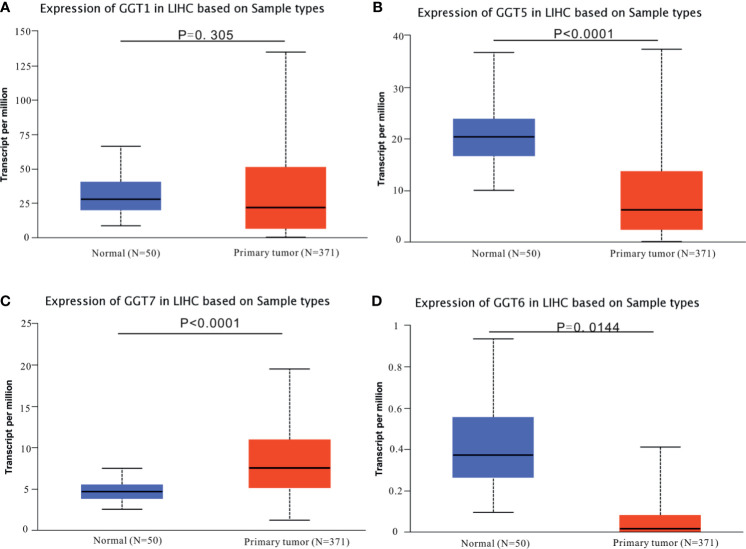
Expression profiles of GGT family genes in HCC specimens and normal tissues from TCGA-LIHC dataset. **(A)** *GGT1*; **(B)** *GGT5*; **(C)** *GGT6*; **(D)** *GGT7*.

The mRNA expression data for GGT family genes (*GGT1*, *GGT5*, *GGT6*, and *GGT7*) from 371 primary HCC samples and 50 normal control samples were analyzed *via* UALCAN web tool. Except for *GGT1* (**Figure 1A**), the other GGT family members (**Figures 1B–D**) were found to be differentially expressed in primary HCC samples (N = 371) and normal control tissues (N = 50). Furthermore, we browsed the cBioPortal webpage to determine the correlations between two GGT family genes (**Figure S1**). Interestingly, a positive relationship was observed not only between *GGT1* and *GGT5* (Spearman coefficient: 0.24, P = 5.143 × 10^−6^) but also between *GGT5* and *GGT6* (Spearman coefficient: 0.38, P = 1.24 × 10^−13^).

### Prognostic Role of GGT Family Genes in HCC

**Figure 2:**
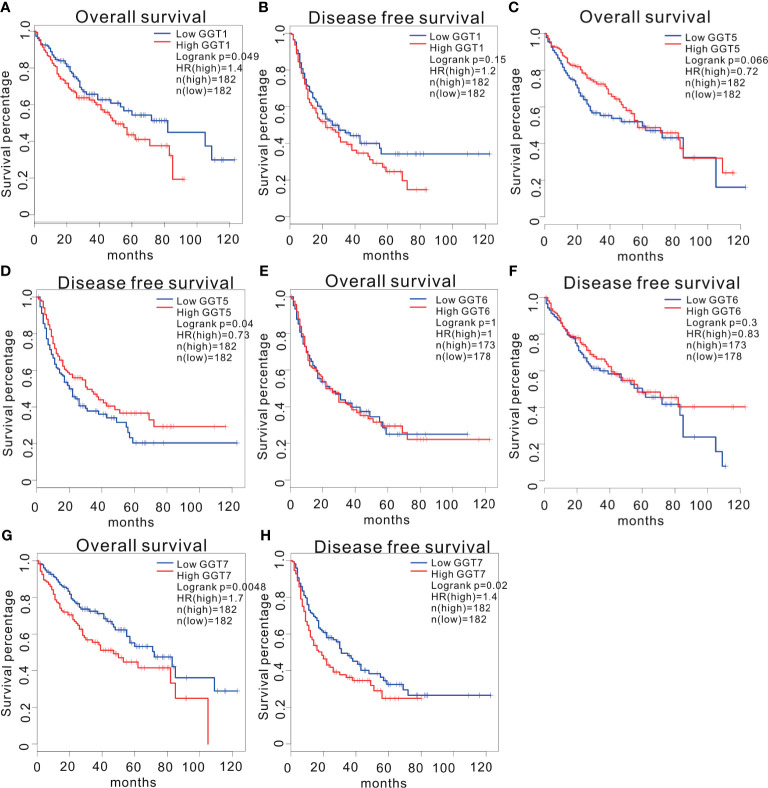
Survival analysis of HCC patients stratified by median expression value of GGT family genes, which were downloaded from GEPIA database. Association between *GGT1* mRNA expression and overall survival **(A)**. Association between *GGT1* mRNA expression and disease-free survival **(B)**. Association between *GGT5* mRNA expression and overall survival **(C)**. Association between *GGT5* mRNA expression and disease-free survival **(D)**. Association between *GGT6* mRNA expression and overall survival **(E)**. Association between *GGT6* mRNA expression and disease-free survival **(F)**. Association between *GGT7* mRNA expression and overall survival **(G)**. Association between *GGT7* mRNA expression and disease-free survival **(H)**.

We utilized GEPIA web tool to determine the prognostic values of GGT family genes in 364 patients with HCC with available follow-up data from TCGA LIHC dataset. A total of 248 patients were males, and 120 were females. The mean age of patients was 59.71 years. A total of 169 patients had TNM I stage, 86 patients had TNM II stage, 85 patients had TNM III stage, and 4 patients had TNM IV stage. Among them, 68 cases were assessed as grade A, 17 cases were assessed as grade B, and no patients were assessed as grade C. The mean follow-up time was 26.44 months. High expression of *GGT1* mRNA was correlated with inferior OS (HR = 1.4, P = 0.049, **Figure 2A**) in patients with HCC; however, this correlation disappeared for DFS (HR = 1.2, P = 0.15, **Figure 2B**). For the *GGT5* mRNA, no positive association was observed between high levels of *GGT5* and worse OS (HR = 0.72, P = 0.066, **Figure 2C**). Fortunately, high expression of *GGT5* was significantly associated with more favorable DFS in patients with HCC (HR = 0.73, P = 0.04, **Figure 2D**). *GGT6* was found to have little prognostic value for OS (HR = 1, P = 1.00, **Figure 2E**) and DFS (HR = 0.83, P = 0.3, **Figure 2F**) in patients with HCC. In addition, high expression of *GGT7* was identified as a promising predictor of both poor OS (HR = 1.7, P = 0.0048, **Figure 2G**) and less favorable DFS (HR = 1.4, P = 0.02, **Figure 2H**) in HCC.

### Prognostic Significance of Serum GGT in Early-Stage HCC

**Figure 3:**
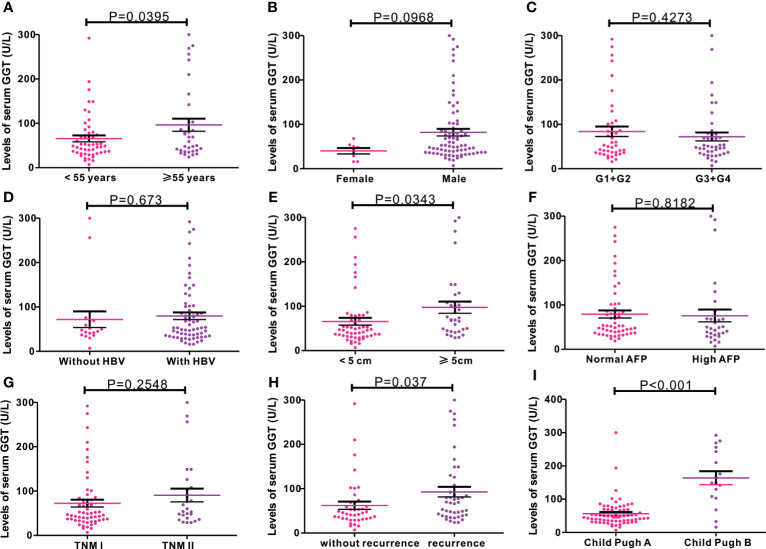
Correlations of serum GGT protein with common clinical variables in 85 cases of early-stage HCC patients from Renmin cohort. **(A)** age (P = 0.0395); **(B)** gender (P = 0.0968); **(C)** G stage (P = 0.4273); **(D)** HBV infection (P = 0.673); **(E)** tumor size (P = 0.0343); **(F)** AFP levels (P = 0.8182); **(G)** TNM stage (P = 0.2548); **(H)** tumor recurrence (P = 0.037); **(I)** Child Pugh grade (P < 0.001).

**Figure 4:**
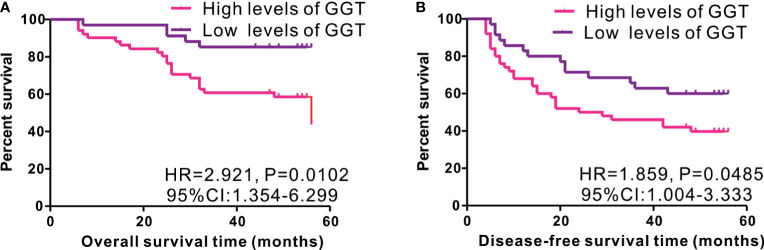
Kaplan-Meier curves of survival in 85 cases of early-stage HCC patients from Renmin cohort stratified by median level of serum GGT protein. High expression of serum GGT protein predicted inferior OS **(A)**, while not for DFS **(B)** in HCC patients with curative hepatectomy.

As serum GGT protein, encoded by *GGT1*, is routinely detected in the clinical setting as a common index reflecting liver injury, we assessed the clinical significance of serum GGT protein in 85 patients with early-stage HCC who received curative hepatectomy. Eight patients were females, and 77 patients were males. The mean age of patients was 52.27 years. A total of 26 cases were defined as TNM I stage, while 59 cases were identified as TNM II stage. The mean follow-up time was 43.13 months. As shown in **Figure 3**, high levels of serum GGT protein were observed in older (age, >55 years) patients with HCC (P = 0.0395), large tumor size (>5 cm, P = 0343), tumor recurrence (P = 0.0037), and Child Pugh grade (P < 0.001). Additionally, we evaluated the prognostic performance of serum GGT protein in patients with early-stage HCC by using Kaplan–Meier curves. As shown in **Figure 4**, patients with early-stage HCC with high levels of serum GGT protein had shorter OS time (HR = 2.921, P = 0.012, **Figure 4A**) and DFS time (HR = 1.859, P = 0.0485, **Figure 4B**) than those with low expression. Finally, Cox regression analysis was performed to determine the independent risk element of early-stage HCC. High expression of serum GGT was identified as a strong prognostic element for inferior OS (HR = 3.114, P = 0.025, (**Table S1**), but not for DFS (HR = 1.198, P = 0.05, **Table S2**) in patients with HCC subjected to curative hepatectomy.

### DNA Methylation, Copy Number, and Mutations in GGT Family Genes

**Figure 5:**
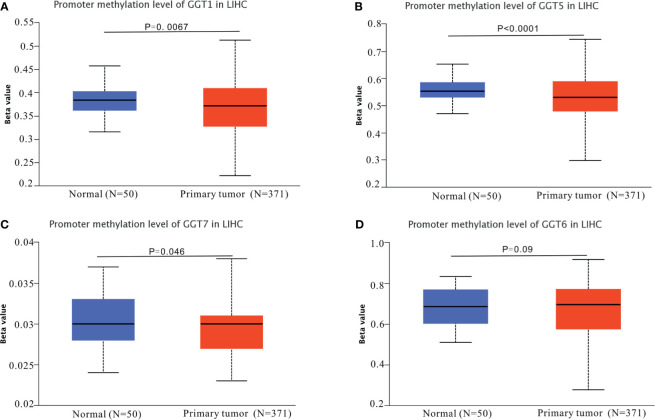
DNA methylation patterns of GGT family genes in HCC tumors and normal controls. DNA methylation of *GGT1* **(A)**, *GGT5* **(B)**, and *GGT7* **(C)** was significantly higher in normal tissues than the corresponding HCC specimens, with the exception of *GGT6* **(D)**.

**Figure 6:**
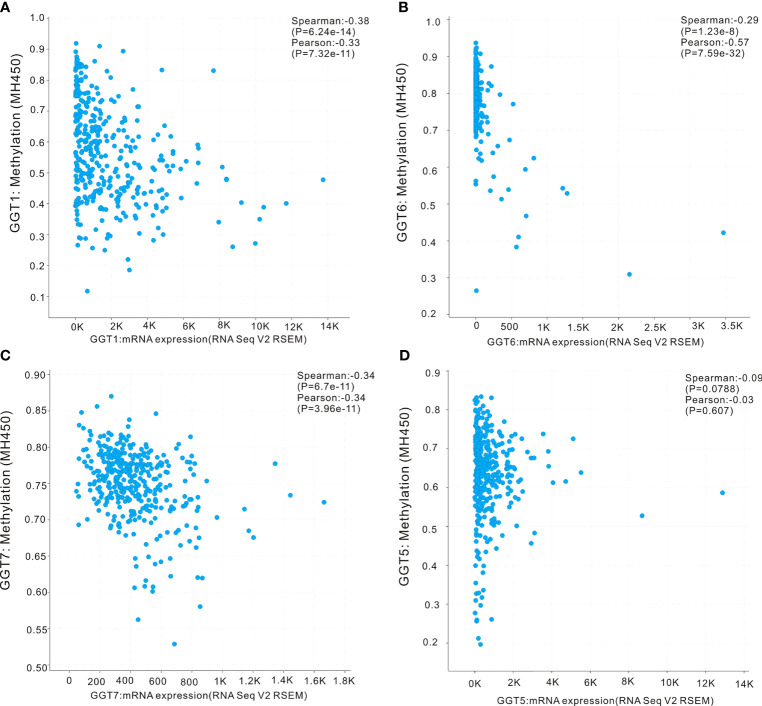
Correlation analysis of DNA methylation with mRNA expression of GGT family members in HCC revealed by cBioPortal webpage. The negative associations between DNA methylation and expression of mRNA were observed in *GGT1* **(A)**, *GGT6* **(B)**, and *GGT7* **(C)**, while this negative relationship disappeared in *GGT5* **(D)**.

First, the UALCAN database was employed to determine whether differential DNA methylation status of GGT family genes existed between liver tumors and normal controls. DNA methylation of *GGT1* (P = 0.0067, **Figure 5A**), *GGT5* (P < 0.0001, **Figure 5B**), and *GGT7* (P = 0.046, **Figure 5C**) was significantly higher in normal tissues than in the corresponding HCC specimens, with the exception of *GGT6* (P = 0.09, **Figure 5D**). The cBioPortal database was also used to determine the potential association between the expression of GGT family genes and DNA methylation. Encouragingly, negative associations between DNA methylation and expression of mRNA were observed for *GGT1* (Spearman coefficient: −0.38, P = 6.24e-14, **Figure 6A**), *GGT6* (Spearman coefficient: −0.29, P = 1.23e-8, **Figure 6B**), and *GGT7* (Spearman coefficient: −0.34, P=6.7e-11, **Figure 6C**); however, this negative relationship disappeared for *GGT5* (Spearman coefficient: −0.09, P = 0.0788, **Figure 6D**). The detailed distribution of CpG sites in GGT family genes in HCC, downloaded from the UCSC Xena database, is listed in **Figure S2**. We also analyzed the copy number status (**Figure S3**) and DNA mutations (**Figure S4**) of GGT family genes in TCGA-LIHC dataset; however, no DNA mutations were found for *GGT6* in TCGA-LIHC dataset.

### Correlations Between GGT Family Genes and Immune Infiltration

**Figure 7:**
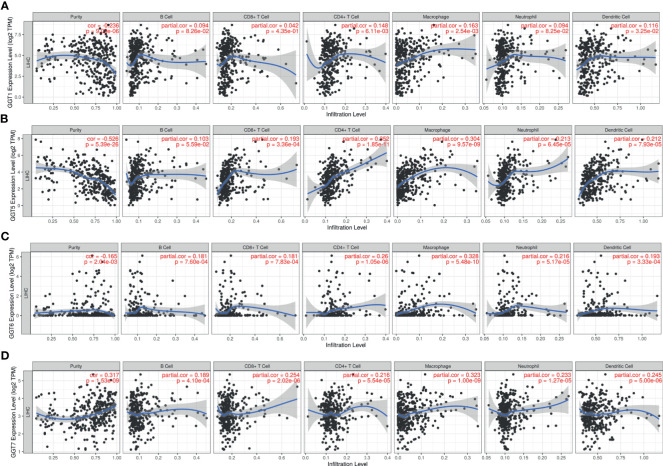
Association between *GGT1* **(A)**, *GGT5* **(B)**, *GGT6* **(C)**, *GGT7* **(D)**, and immune cells (B cell, CD8+ T cell, CD4+ T cell, macrophage, neutrophil, and dendritic cell) in HCC tissues revealed by TIMER database.

TIMER web tool was executed to gain insights into the associations between GGT family genes and infiltration levels of several immune cells in HCC. As shown in **Figure 7A**, expression of *GGT1* was negatively correlated with tumor purity (r = −0.236, P = 9.03e-6) and positively associated with infiltration of CD4+ T cells (r = 0.148, P = 6.11e-3), macrophages (r = 0.163, P = 2.54e-3), and dendritic cells (r = 0.116, P = 3.25e-2). As for *GGT5* (**Figure 7B**), expression of *GGT5* was strongly correlated with tumor purity (r = −0.526, P = 5.39e-26) and positively associated with infiltration of CD8+ T cells (r = 0.193, P = 3.36e-4), CD4+ T cells (r = 0.352, P=1.85e-11), macrophages (r = 0.304, P = 9.57e-9), neutrophils (r = 0.213, P = 6.45e-5), and dendritic cells (r = 0.212, P = 7.935e-5). With regard to *GGT6* (**Figure 7C**), its expression was positively related to CD4+ T cells (r = 0.26, P = 1.05e-6), macrophages (r = 0.328, P = 5.48e-10), neutrophils (r = 0.216, P = 5.17e-5), and dendritic cells (r = 0.193, P = 3.33e-4). Finally, expression of *GGT7* was positively correlated with tumor purity (r = 0.317, P = 1.53e-9), CD8+ T cells (r = 0.254, P = 2.02e-6), CD4+ T cells (r = 0.216, P = 5.54e-5), macrophages (r = 0.323, P = 1.0e-9), neutrophils (r = 0.233, P=1.27e-5), and dendritic cells (r = 0.245, P = 5.0e-6) (**Figure 7D**). Collectively, we found that the four GGT family genes were well correlated with the infiltration levels of immune cells in HCC, especially with CD4+ T cells, macrophages, and dendritic cells.

### Correlations Between GGT Family Genes and Immune Infiltration

**Figure 8:**
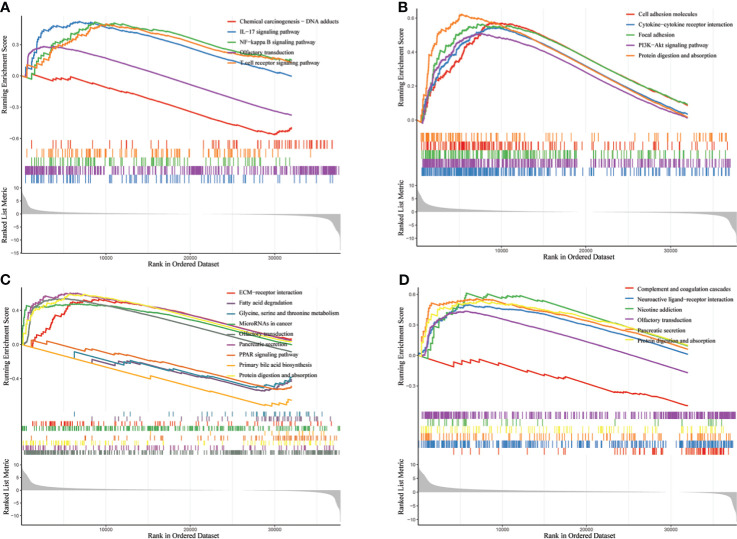
Enrichment analysis of biological pathways of GGT family genes in HCC *via* R software. **(A)** *GGT1*; **(B)** *GGT5*; **(C)** *GGT6*; **(D)** *GGT7*.

TIMER web tool was executed to gain insights into the associations between GGT family genes and infiltration levels of several immune cells in HCC. As shown in **Figure 8A**, expression of *GGT1* was negatively correlated with tumor purity (r = −0.236, P = 9.03e-6) and positively associated with infiltration of CD4+ T cells (r = 0.148, P = 6.11e-3), macrophages (r = 0.163, P = 2.54e-3), and dendritic cells (r = 0.116, P = 3.25e-2). As for *GGT5* (**Figure 8B**), expression of *GGT5* was strongly correlated with tumor purity (r = −0.526, P = 5.39e-26) and positively associated with infiltration of CD8+ T cells (r = 0.193, P = 3.36e-4), CD4+ T cells (r = 0.352, P = 1.85e-11), macrophages (r = 0.304, P = 9.57e-9), neutrophils (r = 0.213, P = 6.45e-5), and dendritic cells (r = 0.212, P = 7.935e-5). With regard to *GGT6* (**Figure 8C**), its expression was positively related to CD4+ T cells (r = 0.26, P = 1.05e-6), macrophages (r = 0.328, P = 5.48e-10), neutrophils (r = 0.216, P = 5.17e-5), and dendritic cells (r = 0.193, P = 3.33e-4). Finally, expression of *GGT7* was positively correlated with tumor purity (r = 0.317, P = 1.53e-9), CD8+ T cells (r = 0.254, P = 2.02e-6), CD4+ T cells (r = 0.216, P = 5.54e-5), macrophages (r = 0.323, P = 1.0e-9), neutrophils (r = 0.233, P = 1.27e-5), and dendritic cells (r = 0.245, P = 5.0e-6) (**Figure 8D**). Collectively, we found that the four GGT family genes were well correlated with the infiltration levels of immune cells in HCC, especially with CD4+ T cells, macrophages, and dendritic cells.

## Discussion

GGT family genes, including *GGT1*, *GGT5*, *GGT6*, and *GGT7*, encode enzymes involved in the metabolism of glutathione and the transpeptidation of amino acids. GGT family genes have been reported to be closely correlated to tumor formation and progression in several malignant neoplasms. In our analysis, we investigated the correlation between the expression of GGT family genes and the survival outcomes of patients with HCC. We demonstrated that the expression levels of *GGT5* mRNA, *GGT6* mRNA, and *GGT7* mRNA were significantly different between normal liver specimens and HCC tissues. Moreover, based on survival analysis, high expression of *GGT7* mRNA was a prognostic factor for less favorable OS and poor DFS in patients with HCC. For the first time, we gained insights into the relationship between GGT family genes and the tumor immune microenvironment and found that GGT family members were positively correlated with several immune cells, such as CD8+ T cells, macrophages, and dendritic cells. To our knowledge, this is also the first study to evaluate the DNA methylation of GGT family genes in HCC.

*GGT1*, a key gene encoding the GGT protein, plays a critical role in maintaining the homeostasis of GSH. Recently, *GGT1* was implicated in the progression and metastasis of several cancers. According to Coradini et al. (23), the expression of *GGT1* was significantly higher in breast tumors than in histologically normal tissues and correlated negatively with BCL2 and positively with TP53. *GGT1* could promote the initiation and progression of clear-cell renal cell carcinoma. Further, its inhibition significantly decreased the migration of tumor cells, suggesting that *GGT1* might be of therapeutic interest for patients with renal cancer (24). Another study further revealed that serum exosomal *GGT1* could be a useful marker for advanced clinical features of patients with renal cancer (25). Kawakami et al. (26) found that the activity of serum exosomal *GGT1* was significantly higher in individuals with prostate cancer than those with benign prostatic hyperplasia. In the present analysis, no differential expression of *GGT1* mRNA was found between liver cancerous specimens and pathological normal tissues; however, low expression of *GGT1* predicted relatively favorable OS in patients with HCC.

*GGT5*, formerly known as GGT-like activity 1 (GGTLA1), plays a critical role in redox regulation, immune function, and drug metabolism (27). Wei and coworkers (12) discovered that *GGT5* was highly expressed in cancer-associated fibroblasts in lung cancer, predicting the poor survival of patients with lung cancer. *GGT5* was also regarded as the key metabolism-related gene in gastric cancer (28) and colon cancer (29). In our study, we found an association between high expression of *GGT5* mRNA and relatively favorable progression-free survival rather than OS. Studies related to *GGT6* in the field of cancer are very few. Nonetheless, two recent studies highlighted the prognostic role of *GGT6* mRNA in head and neck squamous cell carcinoma (30) and papillary renal cell carcinoma (31). However, these situations differ from those in HCC as revealed by our analysis. *GGT7*, formerly known as *GGTL3*, is a novel member of the GGT family. *GGT7* was reported to interact with key proteins associated with the progression of lung cancer (32) and is minimally expressed in gliomas compared to that in normal brain tissues (33). Another study (34) related to *GGT7* and glioblastoma revealed that *GGT7* might play a key role in promoting glioblastoma growth by regulating reactive oxygen species (ROS) levels. To our knowledge, the present study is the first to explore the oncogenic role of *GGT7* in HCC and demonstrated that the expression of *GGT7* mRNA is higher in liver cancerous tissues than in normal liver specimens. Among the four GGT family genes, only the high expression of *GGT7* mRNA was correlated with poor OS and inferior DFS, indicating that *GGT7* might be a promising biomarker for predicting survival in patients with HCC.

GGT protein is a type of membrane-bound enzyme that plays an important role in regulating the production of intracellular glutathione, which is regarded as a classical antioxidant element against ROS (35). In addition, a prior study reported that ROS, a recognized carcinogenic factor, could contribute to the expression of GGT through the redox regulation of various key genes (36). Several preliminary studies have demonstrated that the immune microenvironment could lead to the progression and metastasis of cancer cells through the upregulation of oxidative stress (37–39). Our study also delved into the potential association between GGT family members and the immune microenvironment using the TIMER database. We found that four GGT family members were well correlated with the infiltration abundance of CD8+ T cells, macrophages, and dendritic cells. Biological pathway analysis by GSEA also revealed that *GGT1* was enriched in the T-cell receptor signaling pathway. Abnormal expression of GGT family genes could induce the abnormal production of endogenous ROS, causing cells to be subjected to persistent oxidative stress, subsequently triggering aberrant methylation of the CpG island (6). Consistent with previous findings, the present study also revealed the aberrant CpG island methylation of GGTs between normal liver specimens and liver cancerous tissues. Moreover, a negative relationship was found between the mRNA levels of GGT family genes and DNA methylation. Hence, it is reasonable that elevated serum GGT protein reflects the persistent oxidative stress that is associated with the unfavorable prognosis of patients with HCC.

Sustained oxidative stress by high levels GGT protein would lead to increased risk of gastrointestinal cancer (40), including HCC. Change in activity of GGT is a very useful biomarker in the identification of patients with elevated risk of HCC (41). Moreover, serum GGT combined with other serum biomarkers were effective diagnostic markers of AFP-negative HCC, especially in individuals with early stage, small size or good liver function (42). More researchers continue to assess the prognostic role of serum GGT in cervical cancer (43), esophageal squamous cell carcinoma (44), ovarian cancer (45), renal cell carcinoma (25), and HCC (46). By carrying out a clinical analysis, Zhang et al. (15) found that a high concentration of serum GGT was positively associated with advanced TNM stage and large tumor size and was an independent element for predicting the OS rate of patients with primary HCC. Wang et al. (16) concluded that serum GGT was also an independent risk index for worse OS in individuals with AFP-negative HCC. Another clinical study (47) with 285 patients with HCC who underwent liver transplantation drew a similar conclusion that elevated serum GGT levels were correlated with inferior OS and larger tumor. The strong association between high levels of serum GGT and inferior OS was also observed in patients with HCC subjected to chemoembolization (48). A recent meta-analysis (49) with 9,238 patients with primary HCC demonstrated that preoperative serum GGT protein is a predictive index of unfavorable prognosis for patients with primary HCC. Consistent with the results of previous studies, our analysis based on a cohort of 85 patients with HCC indicated that increased GGT level seems to be associated with aggressive HCC features and is also a strong risk factor for unfavorable OS in patients with HCC. Unlike previous studies, we also determined the association between elevated GGT level and DFS. Unfortunately, this correlation was not observed after multivariate analysis, which might be owing to the limited sample size.

Three limitations of this study must be mentioned. First, the results of this study were mainly derived from systematic bioinformatic analyses. No experimental studies were performed to explore the biological functions of GGT family members. Therefore, basic experiments with regard to GGT family members in HCC cell lines are imminently needed to provide the basis for their clinical application. Besides the limited sample size (N = 85), the clinical study was retrospective and comprised of a single-center cohort. Because of this retrospective nature, we did not obtain serum GGT data at different time points. As a result, we could not assess the dynamic effect of serum GGT, which might be more valuable than that of preoperative serum GGT. Hence, prospective clinical trials with sufficient sample size are also needed to address these issues and promote the clinical utility of GGT protein.

## Conclusion

To our knowledge, this is the first comprehensive analysis of the expression patterns and clinical value of GGT family genes in patients with HCC, providing insights for the experimental exploration of GGT family members as potential targets in HCC. Our study also lays the foundation for the clinical application of the GGT protein in prognostic assessment of patients with HCC.

## Data Availability Statement

The original contributions presented in the study are included in the article/**Supplementary Material**. Further inquiries can be directed to the corresponding authors.

## Ethics Statement

The studies involving human participants were reviewed and approved by Ethics Committee of Renmin Hospital of Wuhan University. The patients/participants provided their written informed consent to participate in this study.

## Author Contributions

WD and XZ designed the research. ST performed the bioinformatic analysis. JL and YG collected the clinical data. ST wrote the manuscript. JL revised the manuscript. All authors contributed to the article and approved the submitted version.

## Funding

This study was supported by the Independent Research Project of Wuhan University (No. 413000342).

## Conflict of Interest

The authors declare that the research was conducted in the absence of any commercial or financial relationships that could be construed as a potential conflict of interest.

## Publisher’s Note

All claims expressed in this article are solely those of the authors and do not necessarily represent those of their affiliated organizations, or those of the publisher, the editors and the reviewers. Any product that may be evaluated in this article, or claim that may be made by its manufacturer, is not guaranteed or endorsed by the publisher.
