# Supplementary material for: Expression Status and Prognostic Significance of Gamma-Glutamyl Transpeptidase Family Genes in Hepatocellular Carcinoma
Source: Front Oncol. 2021 Aug 26;11:731144. doi: 10.3389/fonc.2021.731144 (PMC8426663; doi:10.3389/fonc.2021.731144)
Supplement: Supplementary file 1 [file DataSheet_1.pdf]

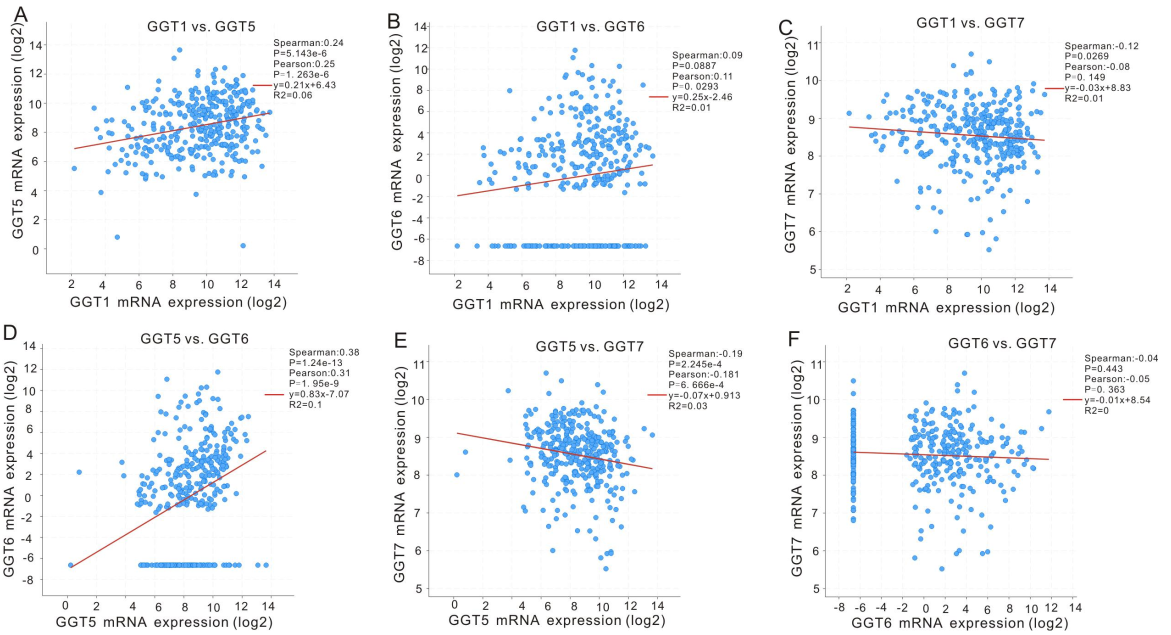

Figure S1. Correlation analysis of GGT family genes in HCC. **A**. GGT1 vs. GGT5; **B**. GGT1 vs. GGT6; **C**. GGT1 vs. GGT7; **D**. GGT5 vs. GGT6; **E**. GGT5 vs. GGT7; **F**. GGT6 vs. GGT7.

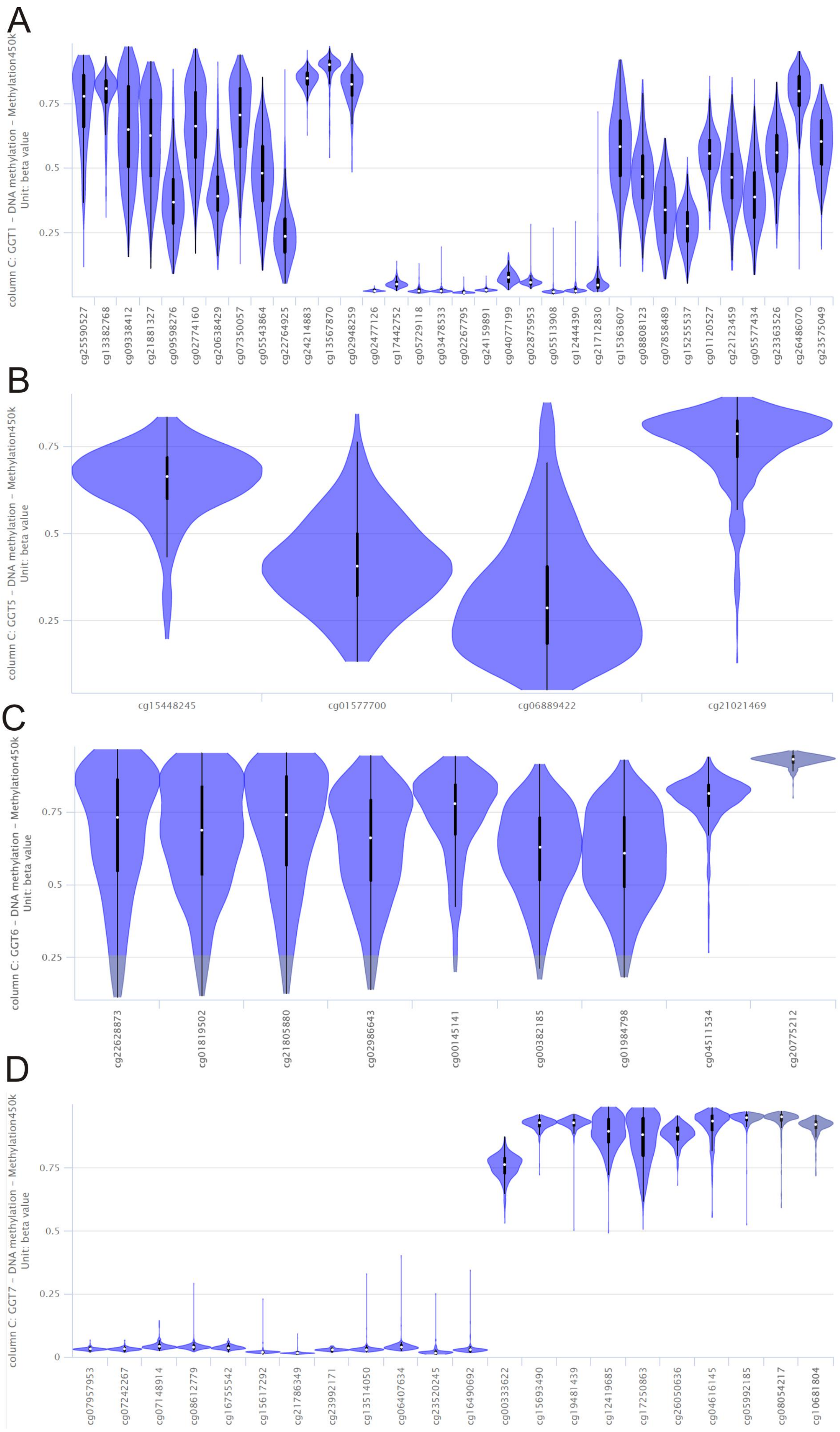
A
column C: GGT1 – DNA methylation – Methylation450k
Unit: beta value
0.75
0.5
0.25
cg25590527
cg13382768
cg09338412
cg21881327
cg09598276
cg02774160
cg20638429
cg07350057
cg05543864
cg22764925
cg24214883
cg13567870
cg02948259
cg02477126
cg17442752
cg05729118
cg03478533
cg02267795
cg24159891
cg04077199
cg02875953
cg05513908
cg12444390
cg21712830
cg15363607
cg08808123
cg07858489
cg15255537
cg01120527
cg22123459
cg05577434
cg23363526
cg26486070
cg23575049
B
column C: GGT5 – DNA methylation – Methylation450k
Unit: beta value
0.75
0.5
0.25
cg15448245
cg01577700
cg06889422
cg21021469
C
column C: GGT6 – DNA methylation – Methylation450k
Unit: beta value
0.75
0.5
0.25
cg22628873
cg01819502
cg21805880
cg02986643
cg00145141
cg00382185
cg01984798
cg04511534
cg20775212
D
column C: GGT7 – DNA methylation – Methylation450k
Unit: beta value
0.75
0.5
0.25
0
cg07957953
cg07242267
cg07148914
cg08612779
cg16755542
cg15617292
cg21786349
cg23992171
cg13514050
cg06407634
cg23520245
cg16490692
cg00333622
cg15693490
cg19481439
cg12419685
cg17250863
cg26050636
cg04616145
cg05992185
cg08054217
cg10681804

Figure S2. The distributions of CpG sites of four GGT family members in HCC. **A**. GGT1; **B**. GGT5; **C**.GGT6; **D**. GGT7.

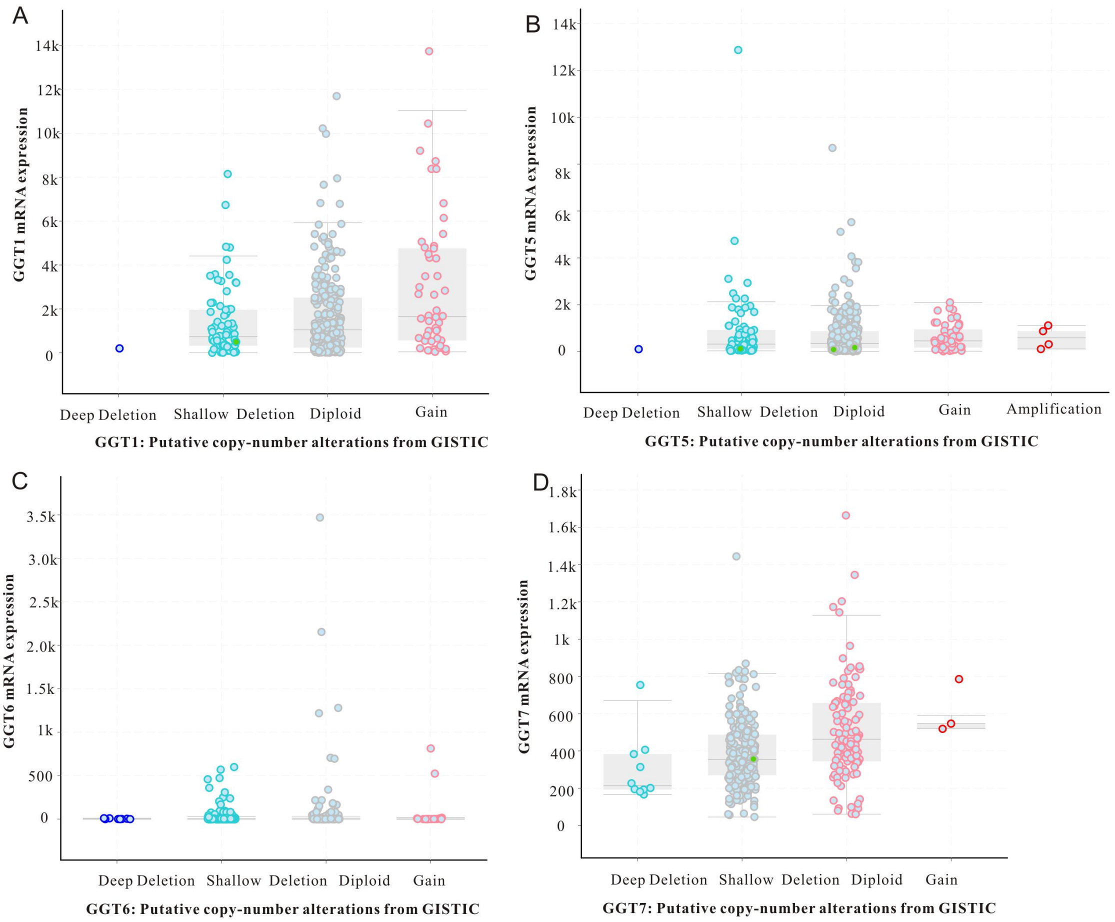

Figure S3. Association between GGT familay genes with copy number in TCGA-LIHC dataset.

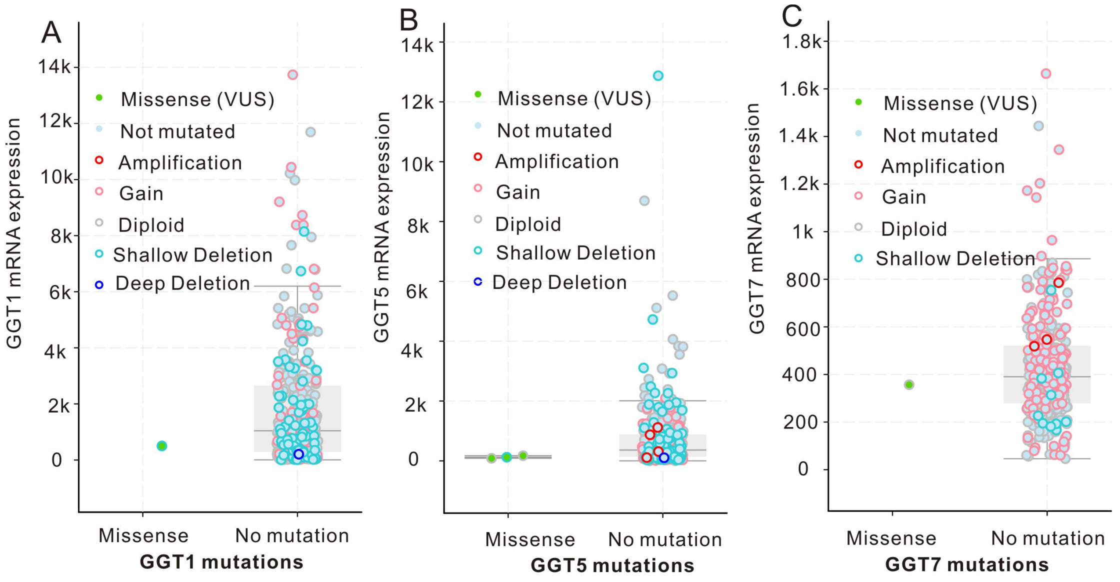

Figure S4. Association between GGT familay genes with mutations in TCGA-LIHC dataset.

Table S1. Cox regression analysis of overall survival in 85 patients with hepatocellular carcinoma

Clinical feature	Univariate Cox regression			Multivariate Cox regression		
	HR	95%CI	P value	HR	95%CI	P value
Gender (Male vs. Female)	23.117	0.125-4643.352	0.236			
Age (≥55 vs. <55)	1.196	0.559-2.558	0.645			
G stage (G3+G4 vs. G1+G2)	2.912	1.225-6.758	0.013	2.534	1.044-6.15	0.04
Tumor size ((≥5 vs. <5cm)	1.977	0.923-4.234	0.08			
TNM stage (II vs. I)	2.812	1.32-5.993	0.007	1.833	0.827-4.065	0.136
HBV infection (Yes vs. No)	1.012	0.404-2.537	0.979			
AFP (High vs. normal)	1.113	0.51-2.432	0.788			
Child Pugh (A vs. B)	0.831	0.335-2.061	0.689			
Serum GGT (High vs. normal)	3.521	1.332-9.31	0.011	3.114	1.115-8.397	0.025

Table S2. Cox regression analysis of disease-free survival in 85 patients with hepatocellular carcinoma

Clinical feature	Univariate Cox regression			Multivariate Cox regression		
	HR	95%CI	P value	HR	95%CI	P value
Gender (Male vs. Female)	1.642	0.508-5.304	0.407			
Age (≥55 vs. <55)	1.936	1.069-3.505	0.029	1.902	1.047-3.455	0.035
G stage (G3+G4 vs. G1+G2)	2.172	1.172-4.024	0.014	2.317	1.244-4.315	0.008
Tumor size ((≥5 vs. <5cm)	1.72	0.951-3.113	0.073			
TNM stage (II vs. I)	1.703	0.927-3.128	0.086			
HBV infection (Yes vs. No)	1.127	0.541-2.345	0.75			
AFP (High vs. normal)	1.011	0.547-1.868	0.973			
Child Pugh (A vs. B)	0.964	0.463-2.006	0.922			
Serum GGT (High vs. normal)	1.974	1.032-3.778	0.04	1.918	1.00-3.681	0.05
